# A synchrotron study of microstructure gradient in laser additively formed epitaxial Ni-based superalloy

**DOI:** 10.1038/srep14903

**Published:** 2015-10-08

**Authors:** Jiawei Xue, Anfeng Zhang, Yao Li, Dan Qian, Jingchun Wan, Baolu Qi, Nobumichi Tamura, Zhongxiao Song, Kai Chen

**Affiliations:** 1 State Key Laboratory for Mechanical Behavior of Materials, Xi’an Jiaotong University, Xi’an, Shaanxi 710049, China; 2 State Key Laboratory for Manufacturing Systems Engineering, Xi’an Jiaotong University, Xi’an, Shaanxi 710049, China; 3 Center for Advancing Materials Performance from the Nanoscale (CAMP-Nano), State Key Laboratory for Mechanical Behavior of Materials, Xi’an Jiaotong University, Xi’an, Shaanxi 710049, China; 4 Advanced Light Source, Lawrence Berkeley National Laboratory, Berkeley, California 94720, USA

## Abstract

Laser additive forming is considered to be one of the promising techniques to repair single crystal Ni-based superalloy parts to extend their life and reduce the cost. Preservation of the single crystalline nature and prevention of thermal mechanical failure are two of the most essential issues for the application of this technique. Here we employ synchrotron X-ray microdiffraction to evaluate the quality in terms of crystal orientation and defect distribution of a Ni-based superalloy DZ125L directly formed by a laser additive process rooted from a single crystalline substrate of the same material. We show that a disorientation gradient caused by a high density of geometrically necessary dislocations and resultant subgrains exists in the interfacial region between the epitaxial and stray grains. This creates a potential relationship of stray grain formation and defect accumulation. The observation offers new directions on the study of performance control and reliability of the laser additive manufactured superalloys.

Single crystalline Ni-based superalloys have been extensively used to manufacture gas turbine blades, blisks, and vane seal segments for aircraft engines and power generating systems^1^,^2^,^3^. The absence of grain boundaries contributes to their outstanding performance when exposed to severe conditions, such as high temperature, vibration, corrosion, and creep rupture^4^. In order to extend the service life and reduce the overall cost of these expensive single crystal blades or vanes, new repair/reshaping techniques are desired, while preserving the single crystalline nature of the Ni-based superalloy^5^. One of the most promising techniques at present is laser additive forming, also known as 3-D printing, laser metal direct forming, or additive manufacturing^6^,^7^. In the repair process via laser additive forming, metal powder is injected into a molten pool formed by controlled laser heating of the substrate. By regulating process parameters, reshaping and manufacturing single crystalline Ni-based superalloy can be realized by directional solidification, in epitaxy with the substrate^8^,^9^. The epitaxial procedure involves preferential columnar dendritic growth that mainly depends on the temperature gradient and solidification velocity. However, due to the influence of solidification kinetics, the preferred orientation sometimes may deviate from the axial direction of the actual growth, and thus stray grains, the crystal orientation of which is different from the substrate and epitaxial grains, are formed during the laser direct forming process^10^,^11^. Two of the major questions that remain to be answered for laser additive forming are how well one can preserve the single crystalline nature and how effectively one can avoid the thermal effects such as hot cracking^5^,^9^. It is therefore important to investigate thoroughly the disorientation between the laser deposited layers and the substrate and the defect density in the laser directly formed materials, which are the main index parameters of epitaxial growth quality and determine the materials resistance to external thermal and mechanical loading.

In previous literature, morphology of the epitaxial and stray grains have been observed using optical and scanning electron microscopy. The crystal orientation has been characterized using electron backscatter diffraction (EBSD) on various Ni-based superalloys^8^,^9^,^12^. High resolution X-ray diffraction (HRXRD) and reciprocal space mapping (RSM) around selected diffraction spots have also been employed to study the disorientation, mosaicity, and lattice mismatch of the laser deposited layers and the substrate^13^,^14^. However, limited by the probe depth of EBSD and the poor spatial resolution of HRXRD and RSM (usually at the scale of hundreds of microns or even millimeters), the orientation and defect distribution and gradient in the laser deposited layers, especially from the substrate to the stray grain region, are not easy to be characterized quantitatively. In this article, synchrotron based white beam X-ray Laue microdiffraction (μXRD) was employed to study a laser additively formed DZ125L Ni-based superalloy, designed in China for application in advanced gas turbine engines^15^. Taking advantage of the micron scale spatial resolution, high orientation resolution, as well as the significant penetration depth of high energy X-ray beam, we studied in depth the microstructural evolution, including both crystal orientation, subgrain boundary distribution, and defect density gradient, over a millimeter size region including the single crystalline substrate, the epitaxial columnar dendrite layers directly formed by laser additive manufacturing, and stray grains. A high density of defects was detected near the epitaxy-stray interface, indicating that the epitaxial-to-stray transition may be related to the defect-assisted heterogeneous nucleation.

## Results

**Figure 1:**
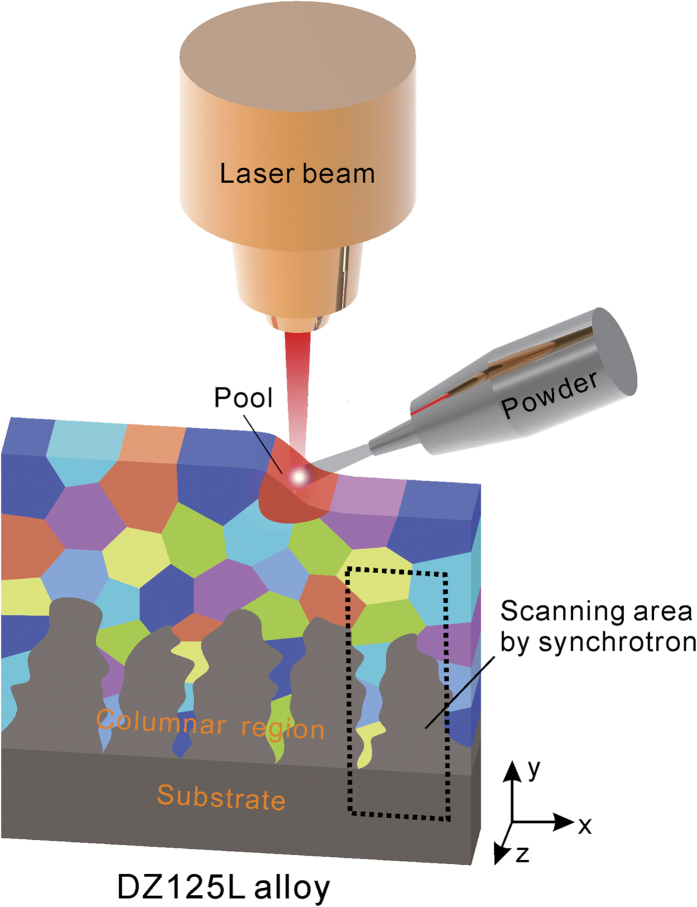
Schematic of laser additive forming experimental setup. A Cartesian coordinate system is built up to represent the sample geometry.

The DZ125L Ni-based superalloy investigated here was rooted from a single crystal of the same material with laser additive processing, as demonstrated in Fig. 1. The chemical composition is listed in Table 1. An area close to the edge of the specimen (indicated by the dashed square in Fig. 1) of 1300 μm (vertically, across the substrate/coating interface) by 500 μm (horizontally) was studied with μXRD, and then etched and investigated under an optical microscope. More experimental details are described in the Methods session.

### Optical microscope investigation

**Figure 2:**
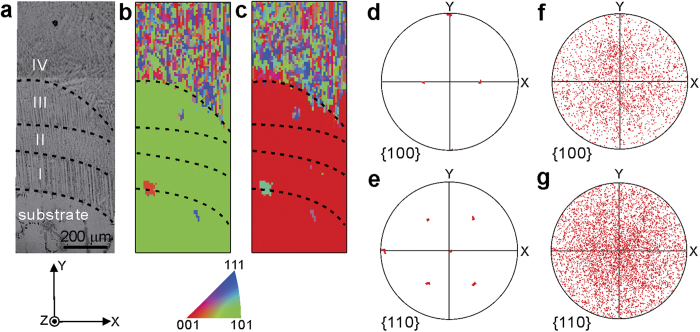
Crystal morphology and orientation of the selected region in the laser additively formed Ni-based superalloy and substrate. (**a**) Crystal grain map obtained under an optical microscope. Four layers are distinguished in the laser deposited materials. (**b**,**c**) Orientation maps of the in-plane **X-** and **Y-**directions obtained from μXRD. Epitaxial and stray grain interfaces are clearly displayed in both maps. (**d**–**g**) {100} and {110} stereographic projection maps of the epitaxial and stray grains, respectively.

The single crystalline substrate is at the bottom of the optical micrograph shown in Fig. 2a. In the laser additively formed alloy on top of the substrate, four zones are distinguished longitudinally, as marked with Roman numerals in the figure. The thicknesses of Zones I and II are uniform across the scanned region, and are measured to be 170 μm and 120 μm, respectively. Zone III, however, is thicker in the region closer to the bulk of the specimen in the left hand side (220 μm) than in the right side (50 μm), which is closer to the edge. Zone IV extends beyond the scanned area. In Zones I and III, columnar dendrites are visible, and their main stems are aligned roughly perpendicular to the substrate/coating interface. In Zone II, however, no clear columnar dendrites are visible in the viewing plane. Dendrites also appear in Zone IV, but their orientation and size are random.

### μXRD results

For an easier representation of crystal orientation, a sample coordinate system **O-XYZ**, as displayed in Figs 1 and 2a, was defined, where the **Z**-axis is perpendicular to the sample surface, the **Y**-axis is along the laser metal deposition direction, and the **X**-axis is normal to both **Y**- and **Z**-axes but within the sample surface. The crystal orientation of the face-centered cubic (FCC) Ni-based superalloy along **X**- and **Y**-directions is color coded in Fig. 2b,c following the most commonly used convention, in which the red, green, and blue colors represent the <100>, <110>, and <111> crystal directions, respectively. The bulk single crystalline substrate at the bottom of these inverse pole figure maps has its 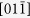, 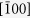, and [011] directions aligned with the **X**-, **Y**- and **Z**-axis, respectively. This is confirmed by its {100} and {110} pole figures, shown in Fig. 2d,e. Polycrystalline grains, which are oriented nearly randomly as indicated by the pole figures shown in Fig. 2f,g, appear on top of the single crystal. In the polycrystalline region, the grains on the right side, which are closer to the edge of the specimen, are as narrow as 10–20 μm in the **X**-direction but elongated to up to 80 μm in **Y**-direction, while most of the grains farther away from the specimen edge are nearly equiaxed, with grain size ranging from 20 to 60 μm. Applying the approached developed previously^16^, we found that most of grain boundaries in this area are ordinary high angle ones, while twinning structures are only detected in a few grains.

Comparing the inverse pole figure maps with the optical micrograph where overlayed dashed curves mark the interfaces between adjacent zones, we conclude that the epitaxial relationship is retained in the first three bottom zones but lost in Zone IV. It is interesting that the crystal grain morphology in Zone II is different from that in Zone I and III but they are all epitaxial. This phenomenon was also observed by other researchers and it is believed to be related to the relative rate of solidification and laser scanning speed, although the detailed mechanism is still unclear^5^.

**Figure 3:**
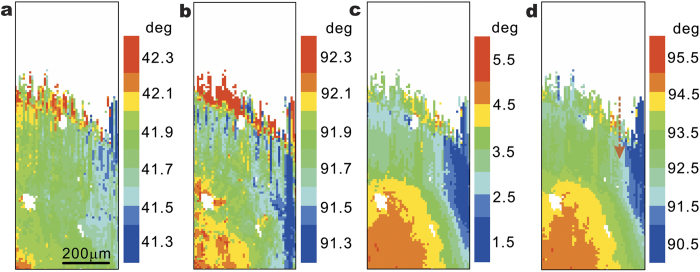
Orientation maps of the epitaxial crystal grain with the angle-axis representation. (**a**) The rotation angle distribution. (**b**–**d**) The projection of rotation axis on the **X-**, **Y-**, and **Z-**axis, respectively. White pixels indicate inclusion in the epitaxial area or the stray grains that epitaxy is missing. High orientation gradient is demonstrated near the interface.

To study the microstructure evolution, the crystalline orientation of the single crystal was plotted in Fig. 3 following the angle-axis representation. Figure 3a shows that the rotation angle over the scanned area distributes in a narrow range between 41.3°–42.3°. From Fig. 3b–d, it is demonstrated that the rotation axis lies almost perpendicular to the **X**- and **Z**-axis and almost parallel to the **Y**-axis. These results agree with the pole figure in Fig. 2 where the 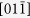, 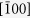, and [011] directions of the single crystal are aligned with **X**-, **Y**- and **Z**-axis, respectively. Moreover, a very homogeneous orientation gradient is observed, from the bottom left, which is inside the substrate to the top right, which is closer to the speci-men edge and the epitaxy-stray interface. Combining the information in both figures, we conclude that the lattice rotates within the single crystal. In the bulk region, the rotation angle is only about 1° over the distance of hundreds of microns. Near the interfacial area, however, the rotation axis is much more random, and the rotation angle is as high as 2° over a length of approximately (150 ± 50) μm.

## Discussion

Bending and twisting of the dendrites have been observed via X-ray radiography technique in conventional single crystal casting, and may eventually result in deleterious sliver defects^17^. Here we have shown that lattice distortion also exists in the epitaxial Ni-based superalloy manufactured by laser additive forming, but the disorientation gradient throughout the bulk part of the scanned 500 μm × 1300 μm area is tiny and almost homogeneous. This is not expected to have significant influence on the mechanical behaviors of the superalloy. However, the inhomogeneous and much higher disorientation at the epitaxy/stray grain interface indicates high residual stress and high density of defects, which may be a nuisance in the laser additive forming repair process, potentially weakening the thermal and mechanical properties of the material, and becoming a reliability issue for the gas turbine blades. Therefore, more efforts are made here to describe the defect distribution in this region.

**Figure 4:**
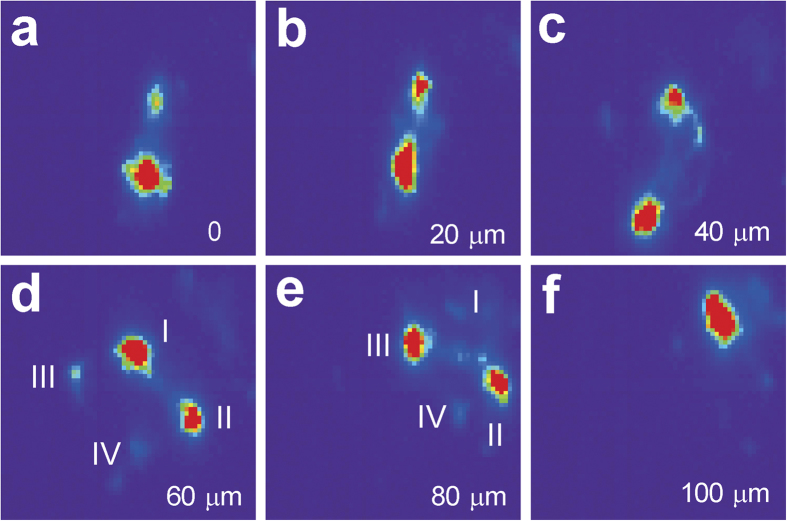
Laue diffraction 022 peak shape snapshot along the vertical brown arrow in Fig. 3 close to the interface. Peak splitting is observed in all patterns less than 100 μm deep. The splitting direction is constant in the range from 0–40 μm, while it varies from 60–80 μm. The reflections remain sharp from 100 μm or deeper.

First we studied the reflection shapes in the Laue patterns taken near the interfacial region. For simplicity, only the 022 reflection of every other Laue diffraction pattern recorded along the dashed line in Fig. 3d in **Y**-direction are displayed in Fig. 4. The diffraction peaks taken in the region from the interface to about 40 μm deep into the epitaxial layer are split into two subpeaks, and within this range in the sample, the relative inclination of the subpeaks with same Miller indices remains almost unchanged. According to the theory of dislocations walls, the subgrains are separated by geometrically necessary boundaries (GNBs) which in turn resulted from the alignment of geometrically necessary dislocations (GNDs), belonging to the same slip system. When going even deeper into the sample, more subpeaks are observed in each Laue pattern, indicating that multiple slip systems are activated, and more than one type of GNDs and GNBs exist in each probed volume of the sample. In the patterns taken at 60 μm and 80 μm away from the interface, at least two pairs of subpeaks are marked, indicating no less than two activated slip systems. Furthermore, comparing these two patterns, the relative intensity of the subpeaks changed dramatically, suggesting that the subgrain volume changed significantly in these two probed volumes. When going even deeper (over 100 μm), sharp diffraction peaks with no streaking or splitting appear again, which is a strong evidence of low defect density and high crystal quality.

**Figure 5:**
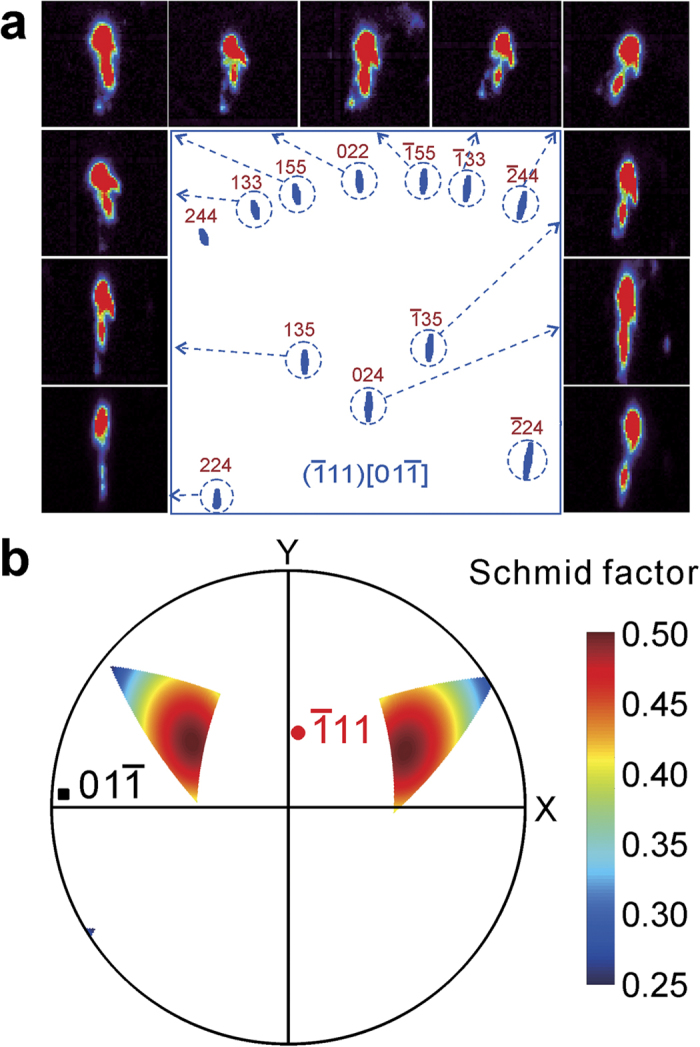
Plastic deformation study for the region less than 40 μm deep. (**a**) Dislocation slip system characterization. Comparing the experimental result with the simulated Laue patterns, the slip system is 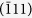 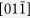. (**b**) Force direction responsible for the dislocation slip. By calculating Schmid factor, the possible force direction is plotted in a polar coordinate system.

**Figure 6:**
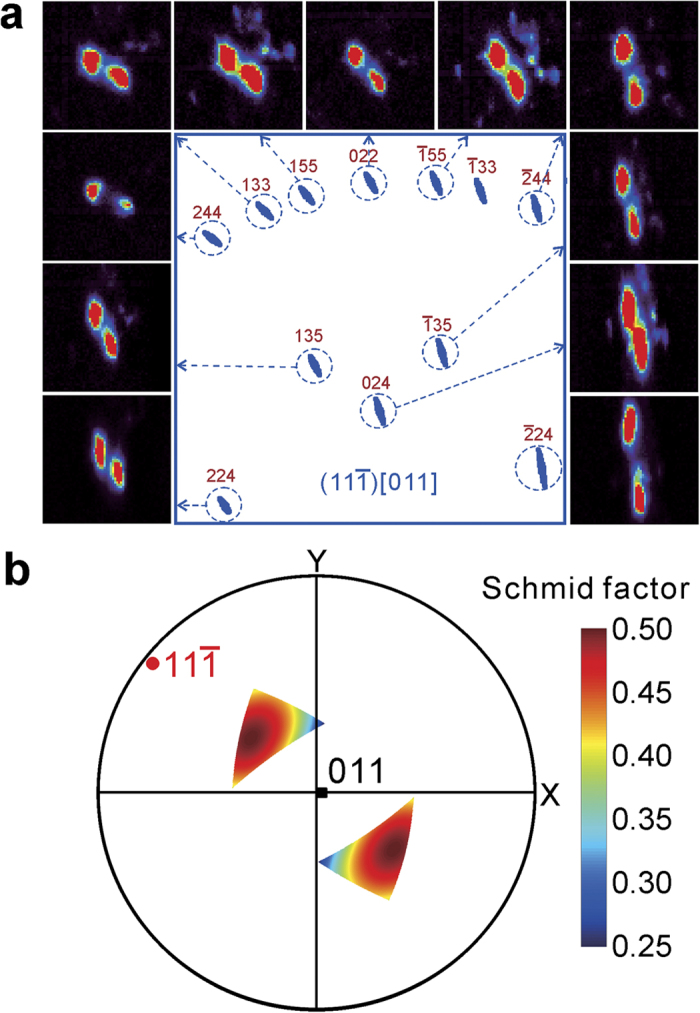
Plastic deformation study for the region from 50 to 90 μm deep. (**a**) One of the multiple activated slip systems, which is identified as 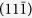 [011]. (**b**) The possible external force component, which is responsible for this slip system, according to Schmid’s law.

We used computer simulations to characterize GNDs and GNBs from the shape of the diffraction peaks. Since the crystal orientation of the single crystal is known from indexing the Laue pattern, the streaking direction of the Laue peaks can be simulated under all the 12 possible {111} 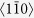 slip systems of FCC Ni-based superalloy, as shown in Figure S1 in supplementary information. By comparing the streaking direction of the reflections in the simulated patterns with the relative tilt directions between each pair of subpeaks in the Laue pattern recorded experimentally, we conclude that the 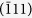 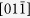 slip system is activated in the range studied from 0–40 μm (shown in Fig. 5a). Based on Schmid’s law^18^, an area is plotted in the pole figure in Fig. 5b to display the probable external loading which has greater resolved shear stress on the activated 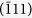 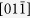 slip system than on any other slip systems by assuming an equivalent uniaxial loading state. For the probed range from 50–90 μm, the results are more complicated because two or even more slip systems are activated. Although it is difficult to identify all the slip systems, we can conclude that the biggest two subgrains at 60 μm are separated by GNDs of the 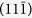 [011] slip system (Fig. 6a), and this slip system is activated throughout this sample range. Similarly, Schmid factor of this slip system is also plotted in the pole figure (Fig. 6b). According to Figs 5b and 6b, the direction of the external force impacting onto the sample varies significantly, and strongly depends on the local microstructure of the specimen.

**Figure 7:**
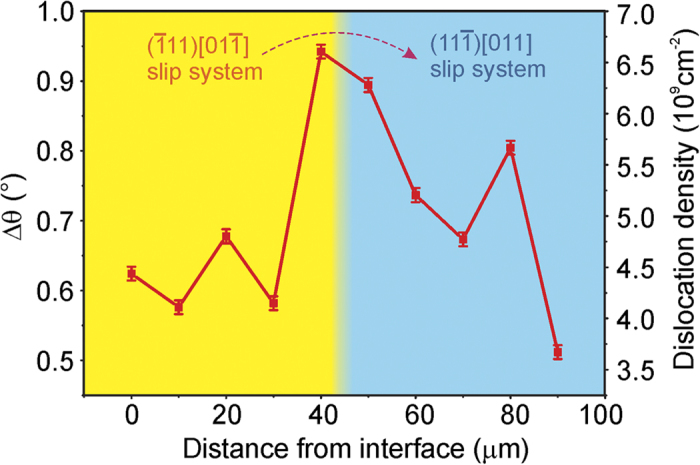
GND density along the pink line shown in Fig. 3. The disorientation angle Δθ is measured from the split subpeaks, and their corresponding GND density is estimated.

The GND density *n* is estimated quasi-quantitatively by measuring the disorientation from the subpeak pairs and applying the following equation:


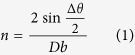


where Δθ, *b*, *D* are the disorientation angle of the subgrains, Burgers vector length, and dislocation wall density, respectively^19^. The GND density is on the order of 10^9^ cm^−2^ (shown in Fig. 7), but could vary by about 100% within the area investigated.

It is worth mentioning that different slip systems are activated at various positions along the interface between the single crystal and the stray grains. 26 Laue diffraction patterns with streaking reflections taken at different **X**-axis positions are analyzed using a similar method as introduced above in Figs 5 and 6. We found that they belong to 9 different slip systems, but we see no evidence that the activated slip system are directly linked to macroscopic sampling position by any straightforward relationship. We believe that this is because the activation of dislocation slip is dependent on the local shear stress status, which, in the laser direct formed sample, is rather complicated and inhomogeneous. This is especially true in the region close to the interface where the cooling rate and temperature gradient are strongly influenced by the specimen geometry and processing parameters. A high quality crystal giving sharp Laue diffraction peaks is observed about 100–150 μm deeper into the sample from the interface, suggesting low density of defects, high homogeneity of the microstructure, and therefore better thermal/mechanical performance of the engineering structure.

There is a significant body of literature on theoretical work and simulations to study the effects of local solidification conditions, such as temperature gradient (*G*) and solidification rate (*V*), on columnar-to-stray transition (CET)^8^,^9^,^20^,^21^,^22^. A greater G or/and smaller V will favor the preservation of the single crystalline structure in the additively formed parts. In the case of rapid solidification conditions, which includes laser additive forming processes, it was proposed that the microstructure will be predominantly columnar when:


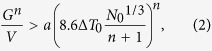


where *a* and *n* are alloy dependent parameter, Δ*T*_0_ the equilibrium liquidus-solidus interval, and *N*_0_ the nucleation density. Although the real experimental condition may deviate from the theoretical assumptions, it is well accepted from Eqn (2) that it is more likely for columnar grains to grow when the nucleation density *N*_0_ is lower, and vice versa, if all other solidification conditions are roughly identical. On the other hand, it has been known that defects in a matrix can act as preferential heterogeneous nucleation sites when the molten material precipitates on it. Introducing defects into the matrix usually increases the number and density of nuclei. If the neighboring nuclei have different crystal orientations, each of them has a good chance to become an individual grain when they grow bigger, and therefore fine grains will form in the product crystal, and the epitaxial relationship with the matrix will be lost.

Furthermore, since the product crystal prefers to nucleate on the matrix coherently to minimize the activation energy as much as possible, the equilibrium shape and shape orientation of the products are strongly dependent on the anisotropic elastic field^23^. Considering that the subgrain boundaries characterized in this study are composed of GNDs of various types and densities from spot to spot along the interface, the elastic field near the interface must be non-uniform and complex, which will lead to precipitates with different preferred morphologies and orientations.

Combining these two effects, inhomogeneously distributed dislocations with various types of slip systems favor the transition from epitaxial growth to stray grains. Although other possibilities and mechanisms cannot be excluded, we speculate that the defects including GNDs and subgrain boundaries detected at the epitaxy/stray interface are responsible for the loss of the single crystalline nature.

In summary, we investigated, with the technique of synchrotron based Laue X-ray microdiffraction, the gradient of crystalline orientation as well as lattice defects of a Ni-based superalloy manufactured via laser additive forming, which is considered to be a promising approach to repair single crystalline superalloy engineering parts. Crystal orientation gradient of about 3° over a sub-millimeter length scale is observed from the substrate to the growth front within the epitaxial regime. Close to the interface where epitaxy is lost, GND density becomes higher, characterized by the streaking and splitting of Laue diffraction peaks, and the dislocation slip system varies from spot to spot along the interface, indicating the inhomogeneous distribution of shear force magnitude and direction. Such micron-scale spatially resolved analysis of defect distribution near the interface unravels possible mechanisms, besides the variation of local temperature gradient and solidification velocity, of why the stray grains are formed after a few layers of epitaxial growth. When the defect density is high, heterogeneous nucleation rate will increase due to the increase of the density of nucleation sites, and thus fine grains tend to form. Moreover, the complex anisotropic elastic field associated with the inhomogeneous defect type and distribution also explains the uneven shape and orientation of the precipitated crystal grains. Therefore, reducing the defect density during epitaxial growth is essential for preventing the formation of high-angle grain boundaries and thus keeping the excellent thermal and mechanical properties of single crystalline superalloy.

## Methods

### Laser additive forming experiment

The laser additive manufacturing was carried out using an independently developed system, XJTU-I, equipped with a neodymium doped yttrium aluminum garnet (Nd:YAG) laser. More detailed information on this system can be found elsewhere^24^. The substrate was made from a cast directionally solidified DZ125L Ni-based superalloy. Powder of the same material and similar composition with spherical shape and a distribution of diameter of 50 ~ 100 μm was injected coaxially by an Ar gas carrier and deposited on the (-100) plane of the single crystalline substrate with the assistance of laser heating. The laser beam spot used in this study was 1.0 mm. The processing parameters used and optimized in previous experiments, were a 4 mm/s laser scanning speed, 230 W laser power, and 9 mm^3^/s powder feeding speed.

### μXRD characterization and analysis

Our μXRD measurement was carried out on Beamline 12.3.2 at the Advanced Light Source (ALS) in Lawrence Berkeley National Laboratory (LBNL) to characterize the microstructure of an area fairly close to the edge of the specimen^25^. In this technique, a high-brilliance synchrotron polychromatic X-ray beam (5–24 keV) was focused to a spot size of about 1 × 1 μm^2^ using a pair of Kirkpatrick-Baez mirrors. The laser deposited sample was mounted on a high resolution x-y scanning stage and tilted 45° relative to the incident X-ray beam. Since the crystalline grain size in the Ni-based alloy was much larger than the micro-focused X-ray beam, single crystal Laue diffraction was used instead of monochromatic polycrystal diffraction. Laue patterns were generated at each scanning position, and recorded in reflection mode with a two-dimensional (2D) DECTRIS Pilatus-1M detector mounted at 90° with respect to the incoming X-ray, approximately 140 mm above the probe spot. An area, which was close to the edge of the specimen (indicated by the dashed square in Fig. 1) and covering the range of substrate, epitaxial laser deposited layers, and stray grains, of 1300 μm along **Y**-direction (perpendicular to the substrate/coating interface) by 500 μm along **X**-direction was scanned with a fixed scanning step size of 10 μm, and at each scanning position the exposure time was 1 s. The resulting 6500 Laue patterns were analyzed using the custom-developed software package XMAS^26^. Diffraction peak positions were determined by fitting each reflection intensity profile with a 2D Gaussian function. The diffraction geometry, including the sample-to-detector distance, the center channel on the detector, and the relative tilts of the detector, was first calibrated by indexing a Laue pattern of a strain-free single crystal silicon chip. All the Laue patterns taken on the specimen were indexed using that same calibration. This approach secures high angular resolution (0.01°) for crystalline orientation, which is important for the evaluation of the quality of single crystals^27^. Furthermore, by studying diffraction peak shapes, information on defects was also obtained, which provides essential clues for predicting the thermomechanical stability of metallic materials^28^,^29^,^30^,^31^.

### Metallographic characterization

After the μXRD experiment, the same laser additive formed specimen was etched using fresh nitro-hydrochloric acid for 5 s and then investigated under an optical microscope to study the evolution of the crystal grain morphology from the substrate through the columnar grain zone and finally to the stray grain zone. Metallographic images were recorded to cover the region that was close to where μXRD study was carried out.

## Additional Information

**How to cite this article**: Xue, J. *et al.* A synchrotron study of microstructure gradient in laser additively formed epitaxial Ni-based superalloy. *Sci. Rep.* **5**, 14903; doi: 10.1038/srep14903 (2015).

## Supplementary Material

Supplementary Information

## Figures and Tables

**Table 1:** Composition of DZ125L superalloy (wt.%).

Material	Co	Cr	W	Al	Ta	Ti	Mo	C	B	Ni
Substrate	9.54	8.74	6.46	5.03	3.96	3.18	2.21	0.12	0.0076	Balance
Powder	9.64	9.70	7.14	4.90	3.78	3.12	2.18	0.09	0.015	Balance
