# Supplementary material for: A synchrotron study of microstructure gradient in laser additively formed epitaxial Ni-based superalloy
Source: Sci Rep. 2015 Oct 8;5:14903. doi: 10.1038/srep14903 (PMC4597195; doi:10.1038/srep14903)
Supplement: Supplementary Information [file srep14903-s1.pdf]

A synchrotron study of microstructure gradient in laser additively formed epitaxial Ni-based superalloy

Jiawei Xue[1], Anfeng Zhang[2], Yao Li[1, 3], Dan Qian[1], Jingchun Wan[1], Baolu Qi[2], Nobumichi Tamura[4], Zhongxiao Song[1], Kai Chen[1, 3]

1. State Key Laboratory for Mechanical Behavior of Materials, Xi’an Jiaotong University, Xi’an, Shaanxi 710049, China
2. State Key Laboratory for Manufacturing Systems Engineering, Xi’an Jiaotong University, Xi’an, Shaanxi 710049, China
3. Center for Advancing Materials Performance from the Nanoscale (CAMP-Nano), State Key Laboratory for Mechanical Behavior of Materials, Xi’an Jiaotong University, Xi’an, Shaanxi 710049, P.R. China
4. Advanced Light Source, Lawrence Berkeley National Laboratory, Berkeley, California 94720, USA

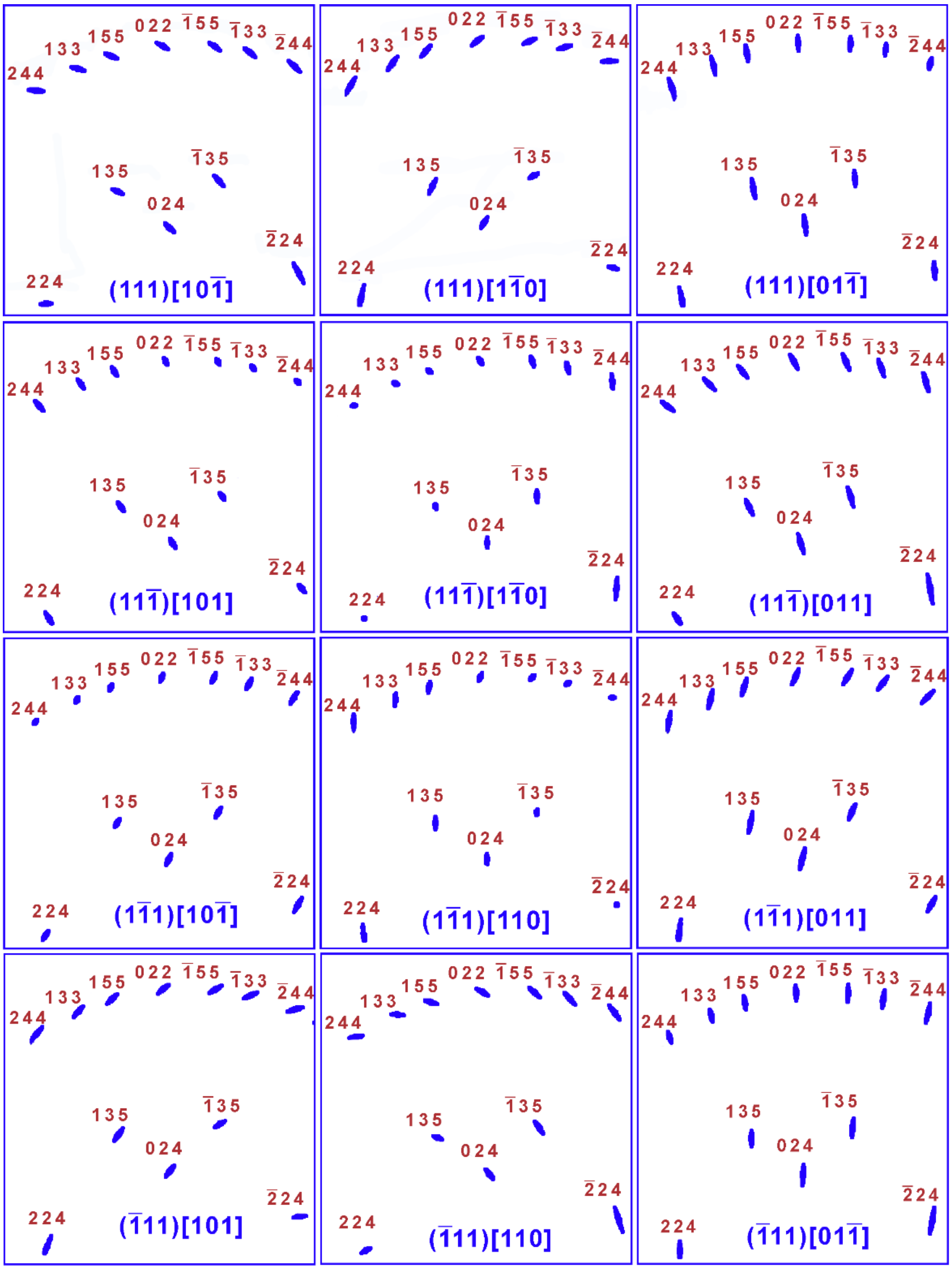

Figure S1 | The simulated Laue diffraction patterns of Ni. All the 12 possible $\{111\} < 1\bar{1}0 >$ dislocation slip systems are considered, and the Ni crystal has the same orientation as the substrate Ni-based superalloy studied in this article.
